# A randomized comparison of gasless laparoscopic appendectomy and conventional laparoscopic appendectomy

**DOI:** 10.1186/1749-7922-9-3

**Published:** 2014-01-08

**Authors:** Bujun Ge, Haibo Zhao, Quanning Chen, Wei Jin, Liming Liu, Qi Huang

**Affiliations:** 1 Department of General Surgery, Tongji Hospital, Tongji University, 389 XinCun Road, Shanghai 200065, PR China

**Keywords:** Gasless laparoscopy, Laparoscopic, Appendectomy

## Abstract

**Introduction:**

The purpose of this study was to compare the clinical outcomes and cost effectiveness of the gasless laparoscopic appendectomy (GLA) and conventional laparoscopic appendectomy (LA).

**Methods:**

From Aug 2010 to Feb 2012, 100 patients with a clinical diagnosis of acute appendicitis in Shanghai Tongji hospital were included in the study and randomly divided into the LA and GLA groups, fifty in the GLA group and 50 in the LA group. The two groups were comparable in age, gender, body mass index, symptom duration, ASA score, and white blood cell count.

**Results:**

The mean surgical duration was 70.6 ± 30.8 min in the GLA group and 62.6 ± 22.0 min in the LA group (*P* = 0.138). The total conversion rate was 8% in the GLA group, while no conversions occurred in the LA group. Postoperative complications did not significantly differ between the two groups. Fentanyl consumption was decreased significantly in the GLA group (*P* = 0.019) postoperatively. The length of the total hospital stay was 4.36 ± 1.74 days in the GLA group compared with 5.68 ± 4.44 days in the LA group (*P* = 0.053). There was a significant decrease in the total hospital cost when the GLA group was compared with the LA group (6659 ± 1782 vs. 9056 ± 2680 Yuan, respectively, *P* < 0.001).

**Conclusion:**

GLA and conventional LA are comparable in terms of operative duration, complications, and total hospital stay. The obvious advantage of GLA is the significantly reduced hospital cost. The demand for postoperative analgesics may also decrease following GLA. In conclusion, GLA is a safe and feasible procedure in selected patients.

**Trial registration:**

Chinese Clinical Trial Register ChiCTR-TRC-10001203.

## Introduction

Since its initial description by Semm in 1983 [1], laparoscopic appendectomy (LA) has been shown to be superior to the open technique and has become the gold standard for the treatment of various types of appendicitis [2]. Compared with the traditional open appendectomy (OA), LA also provides the ability to evaluate the entire peritoneal cavity, making LA preferable for young fertile women for whom the diagnosis of acute appendicitis is difficult, with negative test results for appendicitis in up to 50% of cases [3]. Secondly, LA results in a shorter hospital stay, a quicker return to activity, reduced pain, fewer wound complications, and better cosmesis. Finally, LA is the best choice for obese patients and those with complicated appendicitis, due to improved visualization of the appendix. Despite these advantages, when used in LA, pneumoperitoneum affects cardiopulmonary function [4-6] and is a possible cause of complications, some of which may be severe [7-9]. General anesthesia, which is required to establish CO_2_ insufflation, increases hospital costs and may lead to patient refusal [10]. Therefore, pneumoperitoneum and general anesthesia limit the application of LA, particularly in elderly patients.

To overcome these drawbacks, gasless laparoscopic appendectomy (GLA) was developed in 1993 [11]. GLA was performed without pneumoperitoneum or general anesthesia using various devices to mechanically elevate the anterior abdominal wall with epidural anesthesia. Although a number of potential advantages have been associated with GLA [12], no randomized controlled trial comparing GLA and conventional LA has been reported. The safety and feasibility of this procedure have not been evaluated. Therefore, the purpose of this study was to compare the clinical outcomes and cost effectiveness of the two techniques.

## Materials and methods

This study included 100 patients with a clinical diagnosis of acute appendicitis in Shanghai Tongji hospital between Aug 2010 and Feb 2012. The initial diagnosis was made based on patient history and a physical examination. CT scan was performed in every patient to confirm the diagnosis of acute appendicitis. The patients were randomly allocated into two groups, GLA and LA, using a randomized central computer-generated sequence before they were sent to the operating theatre. With the assumption of a 20% difference in operative duration for the two groups, a minimum sample size of 49 patients per randomization arm was estimated to obtain a power of 80% for detecting this difference at the 5% level. The inclusion criteria were as follows: clinical diagnosis of acute appendicitis, age 15–60 years, American Society of Anesthesiologists Class I or II, informed consent, and willing to abide by the follow-up protocol. The exclusion criteria included the following: 1) serious underlying diseases, patients who could not tolerate the operation and a clear contraindication, 2) obesity (BMI > 28), 3) disease duration longer than 72 hours or appendix abscess, 4) history of previous lower abdominal surgery, 5) refused to receive laparoscopic surgery, 6) mental illness, i.e., could not cooperate under epidural anesthesia, 7) refused to receive general anesthesia, and 8) pregnancy. All of the patients were fully informed about the characteristics of this procedure and its advantages over open or conventional LA. Written consent was obtained from all participants or their family members before surgery. This study was approved by the ethics committee of Shanghai Tongji Hospital, andwas registered with the Chinese Clinical Trial Register (ChiCTR-TRC-10001203).

Two consultant surgeons performed the operations and had sufficient capabilities to perform the two procedures (LA and GLA). Patients who underwent converted GLA were included in the GLA group (intention to treat principle).

The patients in the two groups were managed by the same principles. They were given one prophylactic dose of second-generation cephalosporin just before anesthesia and two doses postoperatively at 8 and 16 h. Antibiotics were continued for a few days only in patients who suffered a perforation. Oral fluids were generally allowed on the day following surgery when bowel sounds returned; however, in some cases, perforation caused ileus and postponed this schedule. When tolerated, a soft diet was introduced. Patient-controlled analgesia (PCA) with intravenous fentanyl was administered as required. The drain, if present, was removed when the aspirate was minimal or nonpurulent, usually in 1 to 2 days. Discharge from the department was done when four conditions were fulfilled: normal body temperature for at least 24 hrs, normal leukocyte count, and passage of a stool, no apparent surgical site infection. The patients were followed up as outpatients for 7 to 10 days and 1 month postoperatively either at the outpatient clinic or by telephone interview.

All of the operative details were recorded. The operative time (minutes) for both procedures was counted from the skin incision to the last skin stitch applied. The parameters evaluated were the duration of the total hospital stay, the hospital cost, the needs for analgesia postoperatively, and the 30-day morbidity.

### Surgical methods

#### GLA group

The patients were advised to void their bladders preoperatively. If unable to do so, a urinary catheter was inserted. After epidural puncture and catheter insertion at T11 ~ T12, continuous epidural anesthesia was administered, and the patients were appropriately medicated according to the block level and surgical requirements. After anesthesia plane satisfaction, the site was prepared with povidone and draped in a sterile manner. Entry into the peritoneal cavity was made by the open method through a 1-cm infraumbilical incision. A 10-mm cannula was then inserted.

**Figure 1:**
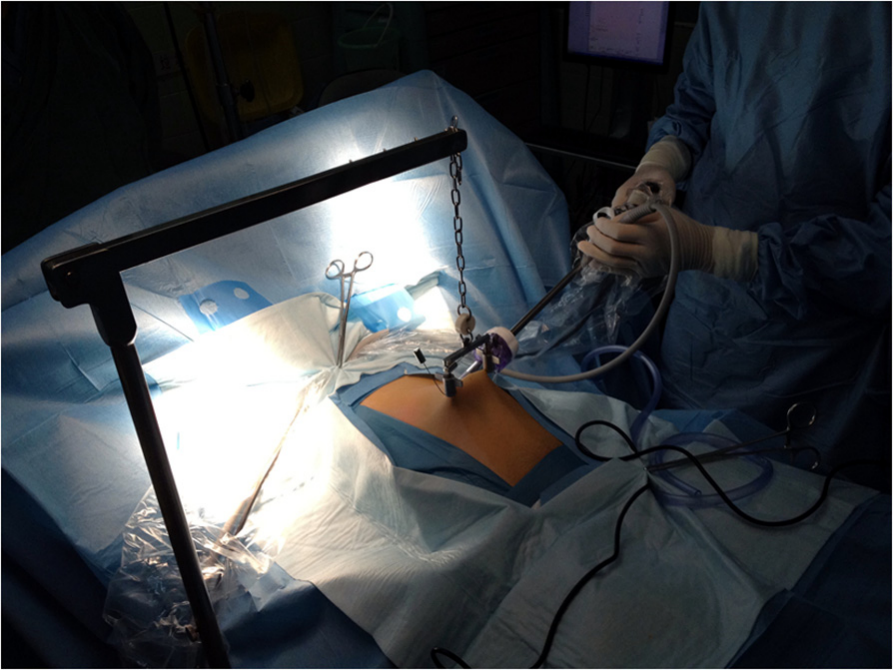
The abdominal wall lifting device and the first trocar.

**Figure 2:**
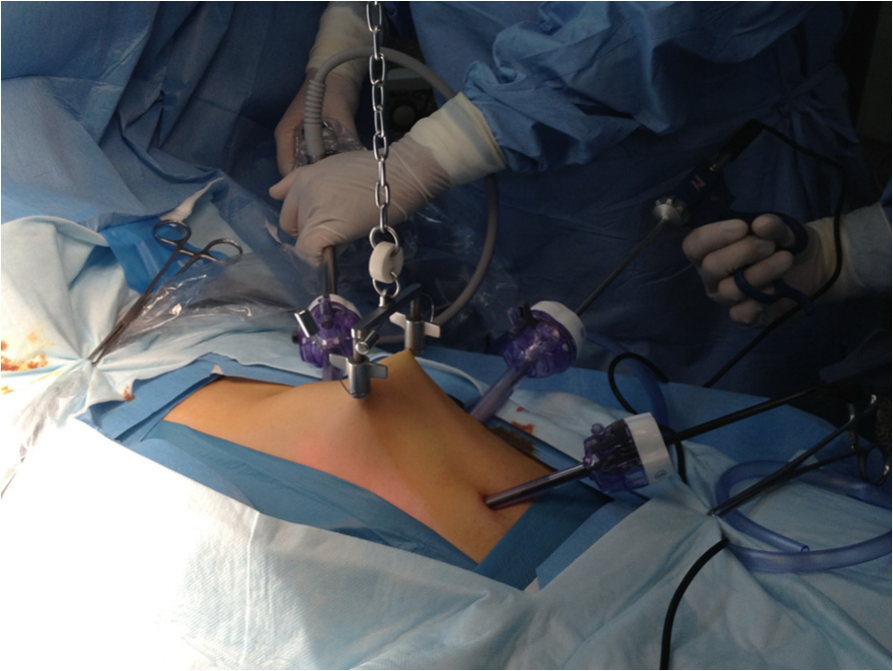
The position of lifting device and all three trocars.

A sterilized stainless steel scaffold consisting of a lifting arm (Mizuho Medical Inc., Tokyo, Japan) was attached to the operating table. The site of needle insertion was first identified in the right iliac zone of the abdomen in the plane of McBurney’s point. One point of needle insertion was near McBurney’s point, and the second insertion site was 6 to 7 cm to the left of it. A sterilized needle (Kirschner wire) was then inserted through the subcutaneous tissue. The abdominal wall was lifted with the needle and fixed to the scaffold using a chain. The lifting blades were attached to the winching retractor, which in turn, was connected to the extension rod (Mizuho Medical Inc., Tokyo, Japan). The lifting system was secured to the side rail of the operative table through the iron side bar. The abdominal wall was pulled up by the winching retractor and then elevated to make a working space as shown in Figures 1 and 2.

A 30° laparoscope was inserted in the supraumbilical port. A general laparoscopic examination of the entire abdomen was performed, including an assessment of the degree of peritonitis from the spread of purulent peritoneal fluid. The lower midline port (5 mm) was then laparoscopically inserted just above the pubic hairline with care not to injure a distended bladder. The left lateral port (10 mm) was inserted at the lateral edge of the rectus muscle equidistant from the other two ports, following transillumination of the abdominal wall by the laparoscope to avoid puncturing the inferior epigastric vessels. This port placement allows the surgeon to operate in a comfortable position with both arms close to their body.

If it became obvious that the appendix was not inflamed, a careful search was performed for other pathology, such as cecal diverticulitis, terminal ileitis, Meckel’s diverticulitis, and small bowel mesenteric adenitis as well as salpingitis, ovarian cyst rupture or torsion, and endometriosis in females.

After identification of the appendix, the mesoappendix was coagulated with bipolar diathermy and cut. The base of the appendix was crushed and clipped with a Hem-o-lock clip or ligated using Vicryl 1. The appendiceal specimen was retrieved through the 10-mm left lateral port using an endo-bag. The 10-mm laparoscope was reinserted, and the pus was completely removed using suction. If a perforation was present, a suction drain was placed in the pelvis through the lower port. A final verification for hemostasis and secure placement of the ligature or clip was made. The umbilical wound was closed with a figure-of-eight 0-polyglactin suture, the wounds were cleaned with antiseptic solution, and the skin was closed with subcuticular 4/0 sutures.

#### LA group

The patients were advised to void their bladders preoperatively. They were intratracheally intubated and treated with general anesthesia. Entry into the peritoneal cavity was made by inserting a 10-mm cannula through a 1-cm supraumbilical incision. Carbon dioxide was injected to establish pneumoperitoneum, and the pressure was maintained at 12 mmHg. The sites of puncture and the operation method were the same as those for the GLA group.

### Statistical methods

The data were analyzed using SPSS (version 19.0; Chicago, IL, USA). Continuous variables, such as age, hospital cost, and operative duration, were presented as the mean ± SD, while categorical variables, such as gender and postoperative complications, were expressed as frequencies. Student’s *t* test was used to compare the means of continuous variables, while categorical variables were compared using the chi-square test or Fisher’s exact test, as appropriate. A probability equal to or less than 0.05 (*P* ≤ 0.05) was considered significant.

## Results

A total of 100 patients were analyzed, 50 in the GLA group and 50 in the LA group. The demographic features of both groups are shown in Table 1. The mean age of the patients was 34.64 ± 15.88 years in the GLA group and 35.32 ± 14.94 years in the LA group. The GLA group contained 29 males and 21 females, whereas the LA group had 24 males and 26 females. The two groups were comparable in age, gender, body mass index (BMI), symptom duration, preoperative temperature, ASA score, main comorbidities, and WBC count. The main comorbidities were hypertension and diabetes. One patient in the GLA group had hypothyroidism, and one patient in the LA group had resected bladder cancer.

**Table 1:** Demographic features

	**GLA (50 cases)**	**LA (50 cases)**	**P value**
Age (ys)	34.64 ± 15.88	35.32 ± 14.94	0.995
Sex (male/female)	29/21	24/26	0.316
BMI (kg/m2)	22.90 ± 4.91	23.35 ± 5.38	0.681
Symptom duration (h)	23.02 ± 20.14	24.42 ± 20.82	0.734
T (°C)	37.8 ± 1.0	37.6 ± 0.7	0.297
Preop WBC (*109/L)	12.6 ± 3.7	12.8 ± 4.3	0.783
ASA score			0.317
1	28	23	
2	22	27	
Comorbidity (patients)	10	5	0.161

As shown in Table 2, the mean surgical duration was 70.6 ± 30.8 min for GLA and 62.6 ± 22.0 min for LA (*P* = 0.138). The histological results were comparable between the two groups. The negative appendectomy rates, as confirmed by histopathology, were 2% (1 case) and 4% (2 cases) in the GLA and LA groups, respectively. For these patients, the final diagnoses were bilateral ovarian cysts in the GLA group patient and sigmoid colon inflammation and a bowel mesenteric inflammatory mass in the LA group patients.

**Table 2:** Comparison of the clinical outcomes

	**GLA (50 patients)**	**LA (50 patients)**	**P value**
Operative time (mins)	70.6 ± 30.8	62.6 ± 22.0	0.138
Conversion (patients)			0.117*
Conversion to LA	3	-	
Conversion to OA	1	0	
Pathologic type (patients)			0.829*
Simple	6	5	
Suppurative	31	34	
Gangrenous or perforated	12	9	
Normal	1	2	
Fentanyl consumption (mg)	0.314 ± 0.218	0.568 ± 0.284	0.019†
Complications (patients)			0.400
Intraabdominal abscess	1	1	
Wound infection	1	2	
Abscess and ileus		1	
Total hospital stay (days)	4.36 ± 1.74	5.68 ± 4.43	0.053
Hospital cost (Yuan)	6659 ± 1782	9056 ± 2680	<0.001

*Fisher’s exact test.

†PCA with intravenous fentanyl was administered to 14 patients in GLA group and 15 patients in LA group as required.

The patient with bilateral ovarian cysts in the GLA group was converted to conventional pneumoperitoneum and underwent anoophorocystectomy. An additional 2 cases in the GLA group were converted to conventional LA due to inadequate visualization caused by obesity or poor anesthesia. One patient in the GLA group was converted to an open appendectomy because the appendiceal root was too thick and could not be treated laparoscopically. The total conversion rate was 8% in the GLA group, while no cases were converted in the LA group. One patient in the GLA group suffered from vomiting during the operation and recovered after the common treatment, which did not cause further complications.

The two modalities did not have significantly different rates of postoperative complications. The main complications included abdominal abscess (1 in the GLA group and 2 in the LA group) and infection of puncture site (1 in the GLA group and 2 in the LA group). In addition, one case of paralytic ileus was caused by an abdominal abscess in the LA group. All of these complications were cured by conservative treatment.

PCA fentanyl was administered to 14 patients in the GLA group and 15 patients in the LA group as required. Fentanyl consumption was decreased significantly in the GLA group (*P* = 0.019). The length of the total hospital stay was 4.36 ± 1.74 days in the GLA group compared with 5.68 ± 4.44 days in the LA group, but the difference was not significant (*P* = 0.053). There was a significant decrease in the hospital cost when the GLA group was compared with the LA group (6659 ± 1782 vs. 9056 ± 2680 Yuan, respectively, *P* < 0.001).

## Discussion

The present study showed that the operative duration, complications, and total hospital stay were comparable between GLA and conventional LA. However, GLA significantly reduced the hospital cost.

The laparoscopic approach to appendectomy has gained wide acceptance over the last 30 years. LA offers a lower risk of postoperative infection and a shorter period for full recovery [13]. Furthermore, LA is a preferred technique for suspected or complicated appendicitis [14]. However, pneumoperitoneum, which is required for LA, may cause a series of complications and prevent the use of LA for patients who are unable to tolerate them. For instance, significant metabolic and hemodynamic alterations are associated with the intra-peritoneal insufflation of carbon dioxide [15]. The arterial partial pressure of carbon dioxide and end-tidal carbon dioxide levels increase in a consistent manner. This phenomenon does not present significant difficulties in the majority of healthy patients, but it can seriously complicate the perioperative course of patients with obstructive pulmonary disease [16]. GLA, which was invented by Smith et al. in 1993 to overcome the disadvantages of conventional LA [11]. Gasless laparoscopy employing an abdominal wall-lifting device has been shown to eliminate the adverse cardiopulmonary effects arising from abdominal insufflation [17]. Many retrospective studies reported in the last 20 years have focused on the technical improvement of GLA [18]. However, GLA is not considered an alternative for appendectomy because no RCTs have established its feasibility and safety.

While gasless laparoscopy effectively prevents the complications associated with CO_2_pneumoperitoneum, inadequate visualization restrains its application in complicated surgeries. A previous RCT showed that the gasless laparoscopic procedure was considerably more difficult to perform and required longer operative times [19]. Appendectomy, however, is a relative simple surgery that requires very little room, making it a good candidate for gasless laparoscopy. The present study showed that there was no significant increase in the operative time for GLA when compared to LA. The incidence of complications was also comparable between the two groups. Wound infection and intraabdominal abscess, which occurred in both groups, are the most common complications for appendectomy and are not dependent on CO_2_ insufflation [10]. In the GLA group, special complications that may be associated with decreased operative room in a gasless condition, such as thermal damage to the small bowel, were not observed. The total conversion rate for GLA was 8%, which is also acceptable when considering that the conversion rate of LA to OA varies from 0% to 27% [20]. Evaluating the entire peritoneal cavity is a main advantage of LA, which is also preserved in GLA especially when appendix is not inflamed obviously. The negative appendectomy in this series is quite low (2% in GLA and 4% in LA). A main reason was that CT scan, with a reported sensitivity that may reach 95% and specificity higher than 95% for diagnosis of acute appendicitis [21,22], was routinely used to confirm the diagnosis in our institution. All of these results indicate that the operative exposure provided by the lift system was adequate for most appendectomies. GLA was shown to be a safe and feasible procedure, which is consistent with previous reports [12].

One of the main advantages of gasless laparoscopy is the avoidance of general anesthesia in some surgeries. Patients who are unable to tolerate general anesthesia and pneumoperitoneum may be candidates for GLA. Our results demonstrate that GLA significantly reduced hospital costs when compared with LA. The difference may not only be due to the change of anesthesia from general to epidural but also due to the trend toward a reduced hospital stay for the GLA group. Fifty cases of OA in the same period were selected randomly to evaluate the cost effectiveness of the GLA. The average cost and hospital stay of conventional appendectomy with small incision and spinal anesthesia were 5028 yuan and 4.08 days respectively (data not shown). The difference between the cost of the GLA and OA was mainly due to the laparoscopic equipment charge (around 1500 yuan). The hospital stay of GLA was similar to that of OA. In the present study, the hospital stay of appendectomy was much longer than which was reported in western countries previously [23,24]. The main reason is that the surgeon in China must ensure that there are no complications or treat if any before discharge to reduce the readmission rate.

Decreased postoperative pain perception is a main advantage for LA compared with OA and is thought to be due to the smaller incisions and minimal tissue handling in LA [25]. However, several studies have shown less postoperative morphine use following gasless laparoscopy when compared with conventional laparoscopy [26]. In addition, other studies have demonstrated that low-pressure laparoscopic cholecystectomy significantly decreases the frequency and intensity of postoperative shoulder tip pain and the demand for postoperative analgesics [27,28]. The present study also found less PCA fentanyl use in the GLA group. Based on these results, CO_2_pneumoperitoneum may be the source of postoperative pain. Gasless or low-pressure laparoscopy may further improve the quality of life following surgery.

The operative exposure provided by the lift system differs from that provided by carbon dioxide insufflation. While the distention provided by pneumoperitoneum is dome shaped, the exposure afforded by the retractor resembles a truncated pyramid. The decreased operative space requires a more experienced surgeon and increases the learning curve. This exposure level was not sufficient for morbidly obese patients, men with very strong abdominal muscles, or those without good anesthesia. Abdominal respiration, which is not eliminated by EPA, produces a “tidal” up and down motion in the surgical field in some patients. To avoid injury to the small intestine, some procedures must be performed during the ebb. Furthermore, gasless exposure is generally limited to a specific quadrant of the abdomen, which restricts exploration of the epigastric zone.

It would be befitting to acknowledge the limitations of our study. First, our follow up was limited to 1 month postoperatively. Our aim was to look for early postoperative complications postdischarge. Second, the treatment allocation and clinical outcome assessment were not blinded. Third, fentanyl consumption may not be representative because PCA was only administered to those patients who asked to use it.

## Conclusions

In our study, GLA and LA had comparable operative durations, complications, and total hospital stay lengths. However, GLA significantly reduced the hospital cost. The demand for postoperative analgesics may also decrease following GLA. In conclusion, GLA is a safe and feasible procedure in selected patients. Future studies should assess GLA in elderly patients with chronic obstructive pulmonary disease. It has been demonstrated that laparoscopic surgery is associated with a lower systemic stress response than open surgery, but intraperitoneal carbon dioxide insufflation attenuates peritoneal immunity [29]. Ultrastructural, metabolic, and immune alterations are observed at the peritoneal surface in response to a pneumoperitoneum [30]. Therefore, gasless laparoscopy may preserve peritoneal immunity theoretically. But this also requires confirmation in future studies.

## Competing interests

The authors declare that they have no competing interests.

## Authors’ contributions

ZH wrote the manuscript. GB and CQ carried out the surgery. HQ and LL participated in the design of the study and performed the statistical analysis. JW conceived of the study, and participated in its design and coordination and helped to draft the manuscript. All authors read and approved the final manuscript.
